# Kratom-Induced Psychiatric Decompensation and Paranoid Delusions

**DOI:** 10.7759/cureus.54626

**Published:** 2024-02-21

**Authors:** Matthew Awad, Hugh H Burke, Scott A Oakman

**Affiliations:** 1 Department of Psychiatry & Behavioral Sciences, University of Minnesota Medical School - Twin Cities, Minneapolis, USA; 2 Department of Psychiatry, Regions Hospital, St. Paul, USA

**Keywords:** substance abuse, mitragayna, delusions, psychosis, kratom

## Abstract

Kratom is a plant extract readily available for purchase in the USA. It is known to produce both stimulant and opioid-related effects, predisposing it to abuse. The long-term effects of kratom are poorly understood. In rare cases, serious side effects have been reported. Here, we report a case of a patient with a history of bipolar type schizoaffective disorder presenting with acute onset paranoia and delusions. The patient had been hospitalized seven times previously with psychotic symptoms, with no reported history of paranoid delusional thought content in previous admissions. It was discovered that the patient had been ingesting increasingly large quantities of kratom in the weeks leading up to the admission. It is believed that kratom may be responsible for the novel symptoms contributing to the patient’s acute psychiatric decompensation.

## Introduction

Kratom, or *Mitragyna speciosa*, is a tropical plant extract from Southeast Asia that has been identified on the US Drug Enforcement Administration’s “Drugs of Concern” list but is not a scheduled or controlled substance [1]. The plant has been traditionally used for medicinal and therapeutic purposes for over 150 years in Southeast Asian countries like Thailand and Malaysia [2]. Its usage has become increasingly popular in the United States. Over 660 calls were received by poison centers between 2010 and 2015, demonstrating a tenfold increase in popularity in those five years [3]. Between 2014 and 2019, 3484 kratom-related cases and 23 deaths were reported to poison centers, with case numbers increasing over time [4]. The plant is easily accessible in the US at gas stations, tobacco shops, and over the Internet. People usually ingest it by chewing its leaves, or boiling it in water and taking it as a tea, or as pills, or by smoking or vaporizing the leaves [5]. Consumers are known to use kratom for pain relief, diarrhea, euphoria, depression, and management of opioid withdrawal [6]. However, despite anecdotal and historical evidence supporting the therapeutic effects of kratom, further investigation of its benefits and harmful effects is required.

Psychiatric complications have been linked to kratom use in previous case reports, and the Centers for Disease Control and Drug Enforcement Administration have stated that kratom may cause psychosis or psychosis-like symptoms [5,7]. Despite this, multiple studies have found no relation between kratom use and psychosis. One study found that 4% of regular kratom users in Malaysia reported psychotic symptoms, while 0.5% of users reported schizophrenia in another Malaysian study [8,9]. Conversely, a 2023 systematic review identified six papers consisting of one case series and five case reports that highlighted worsening psychiatric conditions in kratom users; this review further emphasized the need for published literature on this topic [10]. Here, we present a case report of an individual with a previous diagnosis of schizoaffective disorder who presented with decompensation and new-onset paranoid delusions following heavy kratom use. This report addresses the available kratom literature and devises a potential mechanism for the onset of paranoid delusional thought content in its users. We aim to bring attention to the lack of kratom regulation in the legislation, imperfect detection methods, and the continued need for research on its psychiatric effects.

## Case presentation

Presentation

A 35-year-old male with a past medical history of bipolar type schizoaffective disorder, alcohol use disorder, marijuana use, and multiple suicide attempts presented to the emergency department of a local hospital via the sheriff’s department for crisis evaluation and delusions. His initial vital signs were within normal limits. The patient was paranoid and disoriented on admission. The patient was committed to an inpatient psychiatric floor.

According to the patient’s family and social worker, the patient had been hostile and verbally abusive with his father, accused him of stealing large sums of money from him, and had resigned from his job two weeks prior to admission. The patient was on two antipsychotic medications, paliperidone palmitate and lurasidone, as his family noticed worsening symptoms in the week before his paliperidone palmitate injections. His got his last paliperidone palmitate injection 24 days prior to admission, and had been taking his prescribed lurasidone inconsistently. The patient had paranoid delusional thought content regarding his case manager, whom he also believed he was romantically involved with. Furthermore, the patient continuously reported that she was a police officer who told lies about him to hospitalize him and take his cat from him. Collateral information from the case manager and the patient’s parents indicated that large quantities of kratom had been found in his apartment and that he had been using marijuana. The patient’s outpatient psychiatrist also voiced concerns regarding the patient’s increasing consumption of kratom. A urine drug screen was performed and was positive for only marijuana.

Patient history

**Figure 1:**
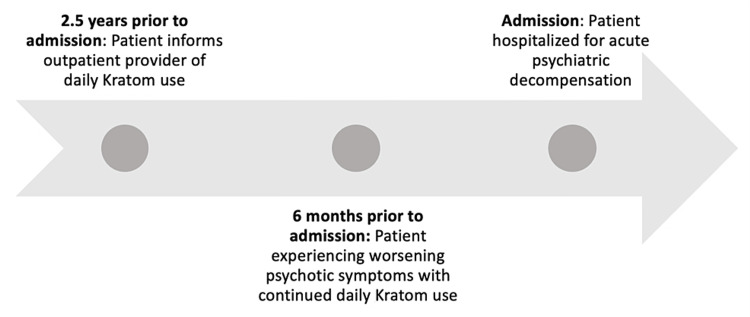
Schematic representation of preceding symptoms and kratom use

In the patient’s seven previous psychiatric hospitalizations, paranoid and delusional thought content was not present. The patient’s case manager reportedly had “never seen him like this,” in reference to the paranoid delusional thought content. Although the patient had been using kratom for two years prior to this admission, his family and case manager had grown increasingly distressed in the weeks leading up to this admission due to a significant escalation in his kratom consumption (Figure 1). Large quantities of kratom were discovered in his residence, and it was discovered that he stopped taking his psychiatric medications for an unspecified amount of time. The patient’s mother reported that he used kratom by putting it in water and drinking it. At the time, the patient informed his mother that he used kratom because it helped with his energy levels.

Notably, the patient’s past medical history included a prior suicide attempt at the age of 20 when the patient put his arm into a woodchipper causing severe orthopedic and soft tissue injuries to the right upper extremity and face requiring multiple surgical procedures. This also caused severe right-eye visual impairment necessitating intense rehabilitation. Additionally, the patient had severe anosognosia regarding his mental health and continuously accused others for the state of his health, including members of his healthcare team and his parents. He persistently denied having a diagnosis of schizophrenia or schizoaffective disorder and would frequently remark on how his situation was the fault of another individual. We believe his visual impairment due to the sequela of his previous suicide attempt and the prominent personality features that accompanied his schizoaffective disorder further contributed to the prolongation of his delusions.

Treatment

Initial treatment included continuation of valproate 1500 mg, olanzapine 15 mg, and paliperidone pamoate 234 mg injection with follow-up injection after three weeks. Olanzapine was discontinued after one day due to possible restless leg syndrome, and lurasidone 60 mg was started. Lurasidone was increased to 120 mg after six days but was eventually discontinued two weeks after admission due to further psychiatric decompensation. Olanzapine 10 mg was initiated after the discontinuation of lurasidone. Three weeks after admission, the patient received a second paliperidone pamoate 234 mg injection, and olanzapine to clozapine cross-tapering was initiated until 250 mg of clozapine, which the patient previously did well on. Unfortunately, the patient continued to experience paranoid delusions and did not show clinical improvement despite seven weeks of antipsychotic treatment. The patient was discharged to an intensive residential treatment service (IRTS).

## Discussion

In the presented case, kratom was believed to be a primary contributor to the patient’s psychiatric decompensation, along with medication nonadherence. The onset of paranoid delusional thought content causing this hospitalization was a novel feature of his schizoaffective disorder and was a persistent symptom throughout his hospital stay despite multiple different anti-psychotic medication trials. The patient was a chronic cannabis user for over 10 years; thus, the temporal nature of his increased kratom use was interpreted as a larger contributor to his decompensation than cannabis use, though it is difficult to ascertain the effect cannabis played in the manifestation of his symptoms.

Kratom’s psychoactive compounds, 7-hydroxymitragynine and mitragynine interact with mu-opioid receptors, providing stimulant effects at low doses and sedative effects at higher doses [7,11]. These effects are highly variable across individuals but can present with a multitude of adverse effects. In a review study that included 2312 kratom exposure cases reported to the National Poison Data System, and a review of records from a medical examiner’s office in New York State, kratom ingestion was found to cause agitation, tachycardia, vomiting, and confusion [12]. Serious side effects like seizure, withdrawal, hallucinations, respiratory depression, coma, and cardiac/respiratory arrest were also reported.

Certain drugs have been shown to be associated with paranoid thought content in psychotic patients, namely, cocaine and other stimulants [13-15]. Although kratom activates mu-opioid receptors, it also has a dose-dependent effect as described above, with stimulant effects at low doses and sedative effects at higher doses [7,11]. For the presented case, we speculate that the patient primarily experienced the stimulant effects of kratom, as he used it to increase his energy. If true, this could be an explanation for the onset of paranoid delusional thought content in this episode of decompensation. Following this theory, kratom acted as a stimulant similar to cocaine, adding novel paranoid delusions to the patient’s current diagnosis of schizoaffective disorder. Unfortunately, there has not been a clinical trial performed on kratom to ascertain the proper dosage of the drug or its effects at various doses [16]. Additionally, effects can vary greatly on an individual basis and there have not been documented animal models explaining kratom’s stimulant-like effects [7]. This is further confounded by a lack of knowledge regarding the amount of kratom that the patient used.

Kratom does not appear on most hospital drug screens [17]. Lu et al. developed a liquid chromatography tandem mass spectrometry procedure to detect the mitragynine alkaloid of kratom, but this has not been widely performed [18]. In our patient, only marijuana was positive on a urine drug screen and further mass spectrometry was not conducted. Hospitals may consider adopting procedures to screen for kratom, as this can guide clinical practice and decisions regarding discharge.

## Conclusions

This case report highlights the psychoactive properties and psychiatric decompensation associated with the use of kratom. Additionally, the onset of paranoid delusions was seen and attributed to kratom due to its stimulant-like effects on users, although it is hard to be certain due to multiple confounding factors such as inconsistent antipsychotic use and concurrent drug use. Kratom use has risen over the last decade and will likely continue rising. If the United States intends to keep kratom legal and easily accessible, further research is imperative to investigate both the benefits and risks of kratom. As always, individuals with psychiatric illnesses should be warned of the potential adverse effects of taking any drug, especially when little is known about its risk profile.
